# Mfsd8 Modulates Growth and the Early Stages of Multicellular Development in *Dictyostelium discoideum*

**DOI:** 10.3389/fcell.2022.930235

**Published:** 2022-06-09

**Authors:** Shyong Quan Yap, William D. Kim, Robert J. Huber

**Affiliations:** ^1^ Environmental and Life Sciences Graduate Program, Trent University, Peterborough, ON, Canada; ^2^ Department of Biology, Trent University, Peterborough, ON, Canada

**Keywords:** Batten disease, CLN7, *Dictyostelium discoideum*, growth, development, lysosome, MFSD8, neuronal ceroid lipofuscinoses

## Abstract

MFSD8 is a transmembrane protein that has been reported to transport chloride ions across the lysosomal membrane. Mutations in *MFSD8* are associated with a subtype of Batten disease called CLN7 disease. Batten disease encompasses a family of 13 inherited neurodegenerative lysosomal storage diseases collectively referred to as the neuronal ceroid lipofuscinoses (NCLs). Previous work identified an ortholog of human MFSD8 in the social amoeba *D. discoideum* (gene: *mfsd8*, protein: Mfsd8)*,* reported its localization to endocytic compartments, and demonstrated its involvement in protein secretion. In this study, we further characterized the effects of *mfsd8* loss during *D. discoideum* growth and early stages of multicellular development. During growth, *mfsd8* ^−^ cells displayed increased rates of proliferation, pinocytosis, and expansion on bacterial lawns. Loss of *mfsd8* also increased cell size, inhibited cytokinesis, affected the intracellular and extracellular levels of the quorum-sensing protein autocrine proliferation repressor A, and altered lysosomal enzyme activity. During the early stages of development, loss of *mfsd8* delayed aggregation, which we determined was at least partly due to impaired cell-substrate adhesion, defects in protein secretion, and alterations in lysosomal enzyme activity. Overall, these results show that Mfsd8 plays an important role in modulating a variety of processes during the growth and early development of *D. discoideum*.

## Introduction

Batten disease, clinically known as the neuronal ceroid lipofuscinoses (NCLs), is a family of neurodegenerative disorders that affect people of all ages and ethnicities (Mole and Cotman, 2015). Clinical symptoms of the disease include vision loss leading to blindness, seizures, deterioration in motor and cognitive function, and premature death (Schulz et al., 2013). There are 13 different subtypes of NCL, each one resulting from a mutation in a distinct ceroid lipofuscinosis neuronal (*CLN*) gene (*CLN1-8*, *CLN10-14*) (Mole and Cotman, 2015). *CLN* genes encode soluble lysosomal proteins (PPT1/CLN1, TPP1/CLN2, CLN5, CTSD/CLN10 and CTSF/CLN13), lysosomal membrane proteins (CLN3, MFSD8/CLN7, and ATP13A2/CLN12), membrane proteins localizing to the endoplasmic reticulum (CLN6 and CLN8), cytoplasmic proteins (GRN/CLN11 and KCTD7/CLN14), and a protein that localizes to synaptic vesicles (DNAJC5/CLN4) (Cárcel-Trullols et al., 2015). While these proteins have a wide range of localizations and functions, it has been suggested that they function in shared or convergent cellular pathways (Persaud-Sawin et al., 2007; Huber, 2020).

Mutations in major facilitator superfamily domain-containing 8 (*MFSD8*) cause a late-infantile form of NCL called CLN7 disease (Aiello et al., 2009). In mammalian cells, MFSD8 is proteolytically cleaved and has been reported to transport chloride ions across the lysosomal membrane (Siintola et al., 2007; Sharifi et al., 2010; Steenhuis et al., 2010; Steenhuis et al., 2012; Wang et al., 2021). However, how MFSD8 influences cellular processes related to NCL pathology is not fully understood. Previous work using *Mfsd8*-deficient mouse embryonic fibroblasts (MEFs) showed that loss of *Mfsd8* alters the amounts of several soluble lysosomal proteins (Danyukova et al., 2018). In addition, loss of *Mfsd8* in mice affects autophagy, neuronal cell survival, and the size and trafficking of lysosomes (Brandenstein et al., 2016; von Kleist et al., 2019; Wang et al., 2021). Further work in human *MFSD8* knockout HEK293T cells revealed the role of MFSD8 in regulating lysosomal chloride conductance, luminal calcium content, lysosomal membrane potential, and lysosomal pH (Wang et al., 2021).

The social amoeba *D. discoideum* is a eukaryotic microbe that is used as a biomedical model organism for studying a variety of human diseases, including the NCLs (Huber, 2021; Kirolos et al., 2021; Mathavarajah et al., 2021; Pain et al., 2021). During the growth phase of the life cycle, haploid amoebae feed on nutrients and divide by mitosis (Mathavarajah et al., 2017). Removal or depletion of the food source triggers a 24-h multicellular developmental program that begins with the chemotactic aggregation of cells into multicellular mounds and ends with the formation of fruiting bodies that are composed of terminally differentiated spores held atop slender stalks of terminally differentiated stalk cells (Mathavarajah et al., 2017). As a result, *D. discoideum* can be used to study a variety of fundamental cellular and developmental processes. The *D. discoideum* genome encodes homologs of 11 of the 13 CLN proteins (Huber, 2016). Recent work on the *D. discoideum* homologs of human tripeptidyl peptidase 1 (TPP1)/CLN2 (Tpp1), CLN3 (Cln3), and CLN5 (Cln5) has provided valuable new insight into the localizations and functions of these proteins in human cells (Huber and Mathavarajah, 2019; McLaren et al., 2019; Smith et al., 2019; Huber, 2020; Huber et al., 2020a; Huber, 2021; McLaren et al., 2021). The *D. discoideum* homolog of human MFSD8 (gene: *mfsd8*, protein: Mfsd8) is expressed throughout the life cycle and a proteomic analysis revealed that Mfsd8 is present in the macropinocytic pathway (Rot et al., 2009; Journet et al., 2012). Recent work in *D. discoideum* showed that Mfsd8 localizes to endocytic compartments, including acidic intracellular vesicles and late endosomes, influences the secretion of two other CLN protein homologs, Cln5 and cathepsin D (CtsD), and interacts with a diversity of proteins during growth and the early stages of multicellular development (Huber et al., 2020b).

In this study, we further characterized the function of Mfsd8 in *D. discoideum* by assessing the effects of *mfsd8* loss on growth and the early stages of multicellular development. Results presented here support a function for Mfsd8 in cell proliferation, pinocytosis, cytokinesis, protein secretion, lysosomal enzyme activity, aggregation, and cell-substrate adhesion. We then integrated these findings into an emerging model summarizing the known roles of Mfsd8 in *D. discoideum*.

## Materials and Methods

### Cell Lines, Antibodies, and Chemicals

Cell lines were grown and maintained on SM/2 agar with *Klebsiella aerogenes* at 21°C (Fey et al., 2007). Cells were also grown axenically in HL5 medium at 21°C and 150 rpm. For all experiments, cells were harvested in the mid-log phase of growth (1–5 × 10^6^ cells/ml). Cultures were supplemented with 100 μg/ml ampicillin and 300 μg/ml streptomycin sulfate to prevent bacterial growth. AX4, hereafter referred to as WT, was the parental cell line for *mfsd8* ^*−*^, which was purchased from the Genome Wide *Dictyostelium* Insertion (GWDI) bank *via* the Dicty Stock Center (https://remi-seq.org) (Fey et al., 2019; Gruenheit et al., 2021). Blasticidin S hydrochloride (10 μg/ml) was used to select *mfsd8* ^*−*^ cells. HL5 and low-fluorescence HL5 were purchased from Formedium (Hunstanton, Norfolk, United Kingdom). 2-N-morpholinoethanesulfonic acid (MES) was purchased from Fisher Scientific Company (Ottawa, ON, Canada). Rabbit polyclonal antibodies against autocrine proliferation repressor (AprA) and countin (CtnA) were provided as gifts by Dr. Richard Gomer (Brock and Gomer, 1999; Brock and Gomer, 2005). Rabbit polyclonal antibody against calcium dependent cell adhesion protein (CadA) was generated and validated in a previous study (McLaren et al., 2021). Mouse monoclonal anti-α-actinin (47-18-9) and mouse monoclonal anti-discoidin (DscA) (80-52-13) were purchased from the Developmental Studies Hybridoma Bank (University of Iowa, Iowa City, IA, United States) (Stadler et al., 1984). Mouse monoclonal anti-β-actin was purchased from Santa Cruz Biotechnology Incorporated (Dallas, TX, United States). Horseradish peroxidase (HRP)-conjugated secondary antibodies were purchased from New England Biolabs (Whitby, ON, Canada). p-Nitrophenyl-β-D-glucopyranoside (487507) (substrate for β-glucosidase), p-Nitrophenyl-a-D-glucopyranoside (487506) (substrate for α-glucosidase), 4-Nitrophenyl-α-D-mannopyranoside (N2127) (substrate for α-mannosidase), *o*-Nitrophenyl-β-D-galactopyranoside (48712-M) (substrate for β-galactosidase), 4-Nitrophenyl-α-D-galactopyranoside (N0877) (substrate for α-galactosidase), 4-Nitrophenyl N-acetyl-β-D-glucosaminide (N9376) (substrate for N-acetylglucosaminidase, NAG), Ala-Ala-Phe-7-amido-4-methylcoumarin (A3401) (substrate for TPP1), Fluorogenic Cathepsin B Substrate III (219392) (substrate for cathepsin B, CTSB), and β-glucosidase (49290) were purchased from Sigma Aldrich Canada (Oakville, ON, Canada). 4-Methylumbelliferyl 6-thio-Palmitate-β-D-Glucopyranoside (19524) (substrate for palmitoyl-protein thioesterase 1, PPT1) and Fluorogenic Cathepsin F Substrate (80350-BP) (substrate for cathepsin F, CTSF) were purchased from Cedarlane Laboratories (Burlington, ON, Canada). The Cathepsin D Activity Assay Kit (10013-596) was purchased from VWR International (Mississauga, ON, Canada).

### Cell Proliferation, Pinocytosis, Cell Size, and Cytokinesis Assays

To assess cell proliferation, cells in the mid-log phase of growth were washed thrice with fresh HL5. Cells were then diluted to 1–2 × 10^5^ cells/ml in HL5 and incubated at 21°C and 150 rpm. Cell concentrations were measured every 24 h over a 96-h growth period using a hemocytometer. The pinocytosis assay was conducted using a method described elsewhere (Rivero and Maniak, 2006; Huber et al., 2014). Briefly, cells in the mid-log phase of growth were placed in 5 ml of HL5 at a density of 5 × 10^6^ cells/ml. 100 μl of a 20 mg/ml fluorescein isothiocyanate (FITC)-dextran (70,000 M_r_) stock solution was added to the 5 ml suspension, which was then incubated for 120 min at 21°C and 150 rpm. 500 μl were collected every 15 min, washed twice with ice-cold Sorenson’s buffer (2 mM Na_2_HPO_4_, 14.6 mM KH_2_PO_4_, pH 6.0) and lysed with 1 ml of buffer containing 50 mM Na_2_HPO_4_ (pH 9.3) and 0.2% Triton-X. Lysates were added to separate wells of black bottom 96-well plates and fluorescence was measured using a BioTek Synergy HTX plate reader and the following filters (460/40 nm for excitation, 528/20 nm for emission) (BioTek Instruments Incorporated, Winooski, VT, United States). To measure cell area, cells in the mid-log phase of growth were deposited onto a hemocytometer. Cells were imaged with a Nikon Ts2R-FL inverted microscope equipped with a Nikon Digital Sight Qi2 monochrome camera (Nikon Canada Incorporated Instruments Division, Mississauga, ON, Canada). The area of each individual cell was quantified using Fiji/ImageJ (Schindelin et al., 2012). To assess cytokinesis, cells (5 × 10^5^ total) in the mid-log phase of growth were collected and deposited onto coverslips placed inside separate wells of a 12-well dish. Coverslips were then submerged in low-fluorescence HL5 and incubated overnight at 21°C. The following day, cells were fixed in −80°C methanol for 45 min and mounted onto slides with Prolong Gold Anti-Fade Reagent containing DAPI (Fisher Scientific Company, Ottawa, ON, Canada). Cells were then imaged using a Nikon Ts2R-FL inverted microscope equipped with a Nikon Digital Sight Qi2 monochrome camera. For each independent experiment, the number of nuclei within each cell (at least 100) was scored and expressed as a percentage of the total number of cells analyzed.

### Plaque Expansion on Bacterial Lawns

Three full inoculation loops of *K. aerogenes* were collected and resuspended in KK2 buffer (0.7 g/L K_2_HPO_4_ and 2.2 g/L KH_2_PO_4_, pH 6.5). 25 μl of the suspension were then deposited onto SM/2 agar and incubated at 21°C. Two days later, *D. discoideum* cells in the mid-log phase of growth were harvested, washed thrice with KK2 buffer, and resuspended in KK2 buffer to obtain a final concentration of 0.4 × 10^6^ cells/ml. Cells (1 × 10^2^ total) were deposited onto the center of the *K. aerogenes* lawns*.* Plaques were captured at the indicated time points using a Leica EZ4W stereomicroscope equipped with an internal 5MP CMOS camera (Leica Microsystems Incorporated, Concord, ON, Canada). Plaque diameters were quantified using Fiji/ImageJ.

### Autocrine Proliferation Repressor Protein Levels During Growth

Cells from the proliferation assay described above were harvested after 24, 48, and 72 h of growth (Huber et al., 2014). Cells were lysed with buffer containing 50 mM Tris–HCl (pH 8.0), 150 mM NaCl, 0.5% NP40, and a protease inhibitor tablet (PIA32965) (Fisher Scientific Company, Ottawa, ON, Canada). Samples of conditioned media (CM) were collected and standardized based on cell number (1 × 10^6^ total). Whole cell (WC) lysates and equal volumes of CM were separated by SDS-PAGE and analyzed by western blotting. The following primary and secondary antibodies were used: anti-AprA (1∶1000), anti-β-actin (1∶1000), and HRP-conjugated secondary antibodies (1:2000). Protein bands were imaged using the ChemiDoc Imaging System (Bio-Rad Laboratories Limited, Mississauga, ON, Canada) and quantified using Fiji/ImageJ.

### Aggregation Assay

Aggregation was examined using a method described previously with minor modifications (Huber, 2017). Briefly, cells (6 × 10^6^ total) harvested from the mid-log phase of growth were deposited into separate wells of a 6-well dish. Cells were allowed to adhere to the surface of the dish for 1 h after which time they were washed two times with KK2 buffer, and then starved in 1 ml of KK2 buffer. Cells were imaged at the indicated times with a Nikon Ts2R-FL inverted microscope equipped with a Nikon 10 Digital Sight Qi2 monochrome camera. A conditioned buffer (CB) swap experiment was conducted to test the effect of proteins secreted by WT cells on the aggregation of *mfsd8* ^*−*^ cells (Huber et al., 2017). Briefly, the CB from WT cells starved for 2 h in 1.5 ml of KK2 buffer was collected and spun down to remove any cells present in the buffer. *mfsd8* ^*−*^ cells were submerged in either 1 ml of KK2 buffer (control) or CB collected from starving WT cells. Cells were imaged at the indicated times with a Nikon Ts2R-FL inverted microscope equipped with a Nikon 10 Digital Sight Qi2 monochrome camera. Aggregation was also examined on 0.5% agar/KK2 (Huber and Mathavarajah, 2018). Briefly, cells in the mid-log phase of growth were harvested from HL5, washed two times with KK2 buffer, and plated (1.5 × 10^8^ cells/ml) in 0.5 μl volumes on 0.5% agar/KK2. Cell spots were imaged at 0 and 5 h using a Nikon Ts2R-FL inverted microscope equipped with a Nikon Digital Sight Qi2 monochrome camera. Images were viewed using NIS Elements Basic Research and analyzed using Fiji/ImageJ. The area after 5 h was expressed as a percentage of the area at 0 h to provide a measure of the amount of aggregation.

### Protein Secretion During Aggregation

Cells (8 × 10^6^ total) in the mid-log phase of growth were deposited into 60 mm × 15 mm Petri dishes and allowed to adhere for 1 h, after which time the HL5 was removed. Adherent cells were washed two times with KK2 buffer and then submerged in 4 ml of KK2 buffer for 4 h, after which time the CB was collected and concentrated using an Amicon Ultra-4 centrifugal filter unit (UFC801024) (Fisher Scientific Company, Ottawa, ON, Canada) according to the manufacturer’s instructions. Adherent cells were lysed with NP40 lysis buffer (recipe noted above). SDS-PAGE and western blotting were then used to determine the effect of *mfsd8* loss on the intracellular and extracellular amounts of CtnA, CadA, and DscA. The following primary and secondary antibodies were used: anti-CtnA (1∶1000), anti-CadA (1∶1000), anti-DscA (1:1000), anti-β-actin (1∶1000), anti-α-actinin (1∶1000), and HRP-conjugated secondary antibodies (1:2000). Protein bands were imaged using the ChemiDoc Imaging System (Bio-Rad Laboratories Limited, Mississauga, ON, Canada) and quantified using Fiji/ImageJ.

### Cell-Substrate Adhesion Assay

Cell-substrate adhesion was assessed using a previously described method with minor modifications (Huber et al., 2017; Huber and Mathavarajah, 2018). Briefly, cells (6 × 10^6^ total) were deposited into separate wells of a 6-well dish and allowed to adhere for 1 h after which time they were washed twice with KK2 buffer and starved in 1.5 ml of KK2 buffer for 4 h. After 4 h, cells were shaken at 150 rpm for 30 min. Samples of CB were collected and cells in CB were counted using a hemocytometer to determine cell dissociation. Cells remaining on the dish were also lysed with NP40 lysis buffer (recipe noted above) and protein concentrations of the lysates were quantified using the Qubit Protein Assay Kit (Q33211) and a Qubit 2.0 Fluorometer (Fisher Scientific Company, Ottawa, ON, Canada).

### Enzyme Activity Assays

To assess various enzyme activities, cells were grown in HL5 overnight to confluency in 60 mm × 15 mm Petri dishes (8 × 10^6^ total). Growth-phase cells and cells starved for 4 h in KK2 buffer were then lysed with buffer containing 50 mM MES pH 6.53 and 0.1% NP40, unless stated otherwise (Phillips and Gomer, 2015). Protein concentrations of lysates were quantified using the Qubit Protein Assay Kit and a Qubit 2.0 Fluorometer, and 150 μg of protein was used for the enzyme assays described below. For all enzyme assays, the absorbance and fluorescence values for the experimental samples were corrected using a lysis buffer control.

Lysosomal hydrolases: To assay α-galactosidase activity, lysates were added to 18 μl of 2 mM of 4-Nitrophenyl-α-D-galactopyranoside in sodium citrate/phosphate buffer (pH 4.5) (Kilpatrick and Stirling, 1976). Samples were then incubated for 45 min at 37°C and quenched with equal volume of 1 M sodium glycinate buffer (pH 10.4). The reactions were then deposited into 96-well black clear bottom plates and the absorbance (405 nm) was measured using a BioTek Synergy HTX plate reader. To assay β-galactosidase activity, lysates were added to 36 μl of 25 mM of *o*-Nitrophenyl-β-D-galactopyranoside in 100 mM citrate buffer (pH 4.0) (Maruhn, 1976). The reaction mixtures were incubated for 45 min at 37°C, after which time an equal volume of 2-amino-2-methyl-1-propnanol/HCl solution was added. The reactions were then deposited into 96-well black clear bottom plates and the absorbance (405 nm) was measured using a BioTek Synergy HTX plate reader. To assay α-glucosidase activity, WC lysates were incubated in 2 mM p-Nitrophenyl-a-D-glucopyranoside within 0.1 M sodium succinate (pH 6.0) in a total volume of 150 μl (Wimmer et al., 1997). The reaction solution was incubated for 1 h at 65°C followed by the addition of 300 μl of 1 M sodium carbonate to quench the reaction. The quenched reactions were then deposited into 96-well black clear bottom plates and the absorbance (395 nm) was measured using a BioTek Synergy HTX plate reader. To assay β-glucosidase activity, lysates were added to 18 μl of 10 mM of p-Nitrophenyl-β-D-glucopyranoside in 50 mM acetate buffer (pH 5.0) (Coston and Loomis, 1969). The reactions were incubated at 35°C for 45 min. The reactions were then quenched with an equal volume of 1 M sodium carbonate and deposited into 96-well black clear bottom plates and the absorbance (405 nm) was measured using a BioTek Synergy HTX plate reader. To assay α-mannosidase activity, lysates were added to 18 μl of 5 mM of 4-Nitrophenyl-α-D-mannopyranoside in 5 mM acetate buffer (pH 5.0) (Loomis, 1970). Samples were incubated for 45 min at 35°C after which time an equal volume of 1 M Na_2_CO_3_ was added to quench the reactions. The quenched reactions were then deposited into 96-well black clear bottom plates and the absorbance (405 nm) was measured using a BioTek Synergy HTX plate reader. To assay NAG activity, WC lysates were added to 75 µl of 7 mM of 4-Nitrophenyl N-acetyl-β-D-glucosaminide in 100 mM acetate buffer (pH 5.0). Samples were then incubated for 5 min at 35°C, after which time an equal volume of 1 M Na_2_CO_3_ was added to quench the reactions (Loomis, 1969; Huber and Mathavarajah, 2018). The quenched reactions were then deposited into 96-well black clear bottom plates and the absorbance (405 nm) was measured using a BioTek Synergy HTX plate reader.

Ppt1 and Tpp1: To assay Ppt1 activity, lysates were added to 5 µl of 9 mM of 4-Methylumbelliferyl 6-thio-Palmitate-β-D-Glucopyranoside dissolved in McIlvaine phosphate/citric-acid buffer (pH 4) supplemented with 15 mM dithiothreitol and 0.375% Triton X-100*.* The reactions were incubated at 37°C for 1 h after which time they were boiled for 3 min at 95°C (van Diggelen et al., 1999; Brand et al., 2018). Once the reactions were cooled, 2.75 µl of 2.5 M NaOH and 4 µl of 0.025 U/μl β-glucosidase were added to the reactions and incubated for another hour at 37°C*.* The reaction was quenched by adding 0.5 M sodium carbonate-bicarbonate buffer containing 0.025% Triton X-100 (pH 10.7). The quenched reactions were then deposited into 96-well opaque black bottom plates and the fluorescence was measured using a BioTek Synergy HTX plate reader and the following filters (360/40 nm for excitation, 460/40 nm for emission). To assay Tpp1 activity, cells were lysed with buffer containing 50 mM sodium phosphate (pH 6.5) and 0.5% NP40, and then added to 80 µl of 200 μM Ala-Ala-Phe-7-amido-4-methylcoumarin dissolved in reaction buffer (150 mM NaCl, 100 mM sodium acetate, pH 4.5, 0.1% Triton X-100) (Stumpf et al., 2017). Reactions were incubated in the dark at 37°C for 1 h and then quenched by adding stop solution (150 mM NaCl, 100 mM sodium acetate, pH 4.3). The quenched reactions were then deposited into 96-well opaque black bottom plates and the fluorescence was measured using a BioTek Synergy HTX plate reader and the following filters (360/40 nm for excitation, 460/40 nm for emission).

Cathepsins: To assay CtsB activity, methods were adapted with minor revisions (Barrett, 1980). Briefly, WC lysates were incubated in reaction buffer (352 mM KH_2_PO_4_, 48 mM Na_2_HPO_4_, 4 mM EDTA, pH 6.0) with 8 mM cysteine in a total volume of 150 μl and incubated at 40°C for 5 min. A final concentration of 5 μM of substrate was added to the reaction solution, which was then incubated at 40°C for 30 min. The reaction solutions were quenched with 200 μl of 100 mM sodium chloroacetate (30 mM NaC_2_H_3_O_2_, 70 mM HC_2_H_3_O_2_, pH 4.3). The quenched reactions were then deposited into 96-well opaque black bottom plates and the fluorescence was measured using a BioTek Synergy HTX plate reader and the following filters (360/40 nm for excitation, 460/40 nm for emission). CtsD activity was measured following the manufacturer’s instructions specified in the Cathepsin D Activity Assay Kit. Briefly, cells were lysed with 100 µl of chilled cell lysis buffer (provided in kit) and incubated on ice for 10 min. The solution was then centrifuged for 5 min. Following centrifugation, 27.5 µg of clear cell lysate was added to 100 µl of reaction buffer (provided in kit) mixed with 2 µl of substrate. Reactions were incubated at 37°C for 1 h and 30 min. The reactions were then deposited into 96-well opaque black bottom plates and the fluorescence was measured using a BioTek Synergy HTX plate reader and the following filters (360/40 nm for excitation, 460/40 nm for emission). To assay CtsF activity, WC lysates were added to 0.5 µl of 25% HCl and 1 µl of 250 μg/ml pepsin. 50 µl reactions were incubated for 1 h at 37°C after which time 0.24 µl of 0.5 mM Fluorogenic Cathepsin F Substrate followed by 5.76 µl dimethyl sulfoxide and 70 µl of 0.1 M sodium phosphate buffer containing 1 mM EDTA and 0.1% (v/v) PEG 6000 (pH 6.5) were added to the reaction mixture (total volume of 120 µl). Reactions were incubated at 27°C for 1 h (Fonovic et al., 2004). The reactions were then deposited into 96-well opaque black bottom plates and the fluorescence was measured using a BioTek Synergy HTX plate reader and the following filters (360/40 nm for excitation, 460/40 nm for emission).

### Statistical Analyses

Numeric data are reported as the means ± SEM. Statistical analyses for all experiments were performed using GraphPad Prism 8 (GraphPad Software Incorporated, La Jolla, CA, United States). A one-sample *t*-test was used for evaluating data where the raw data between biological replicates was inherently variable (e.g., quantifying pixel intensity from a western blot). A *p*-value < 0.05 was considered significant for all analyses and n represents the number of independent experiments that were performed. Details on the specific statistical analyses performed are found in the figure captions.

## Results

### Expression Profile of *mfsd8* During the *D. discoideum* Life Cycle

A previous study in *D. discoideum* showed that Mfsd8 localizes to endocytic compartments, linked the function of Mfsd8 to protein secretion, and revealed the Mfsd8 interactome during growth and starvation (Huber et al., 2020b). To gain further insight into the function of Mfsd8 in *D. discoideum*, here, we performed an in-depth characterization of an *mfsd8* ^*−*^ cell line. The expression of *mfsd8* increases during the early stages of development, which involves the chemotactic aggregation of cells into multicellular mounds (Rot et al., 2009; Mathavarajah et al., 2017). Following mound formation, *mfsd8* expression decreases dramatically reaching its lowest level just prior to terminal differentiation of pre-spore and pre-stalk cells. Expression then rises slightly during fruiting body formation. This expression profile suggested to us that Mfsd8 primarily functions during growth and the early stages of development. As a result, we focused our analysis on the effects of *mfsd8*-deficiency on processes that occur during these stages of the life cycle.

### *mfsd8* ^*−*^ Cells Display Increased Proliferation and Accumulation of Fluorescein Isothiocyanate-Dextran During Growth

**FIGURE 1:**
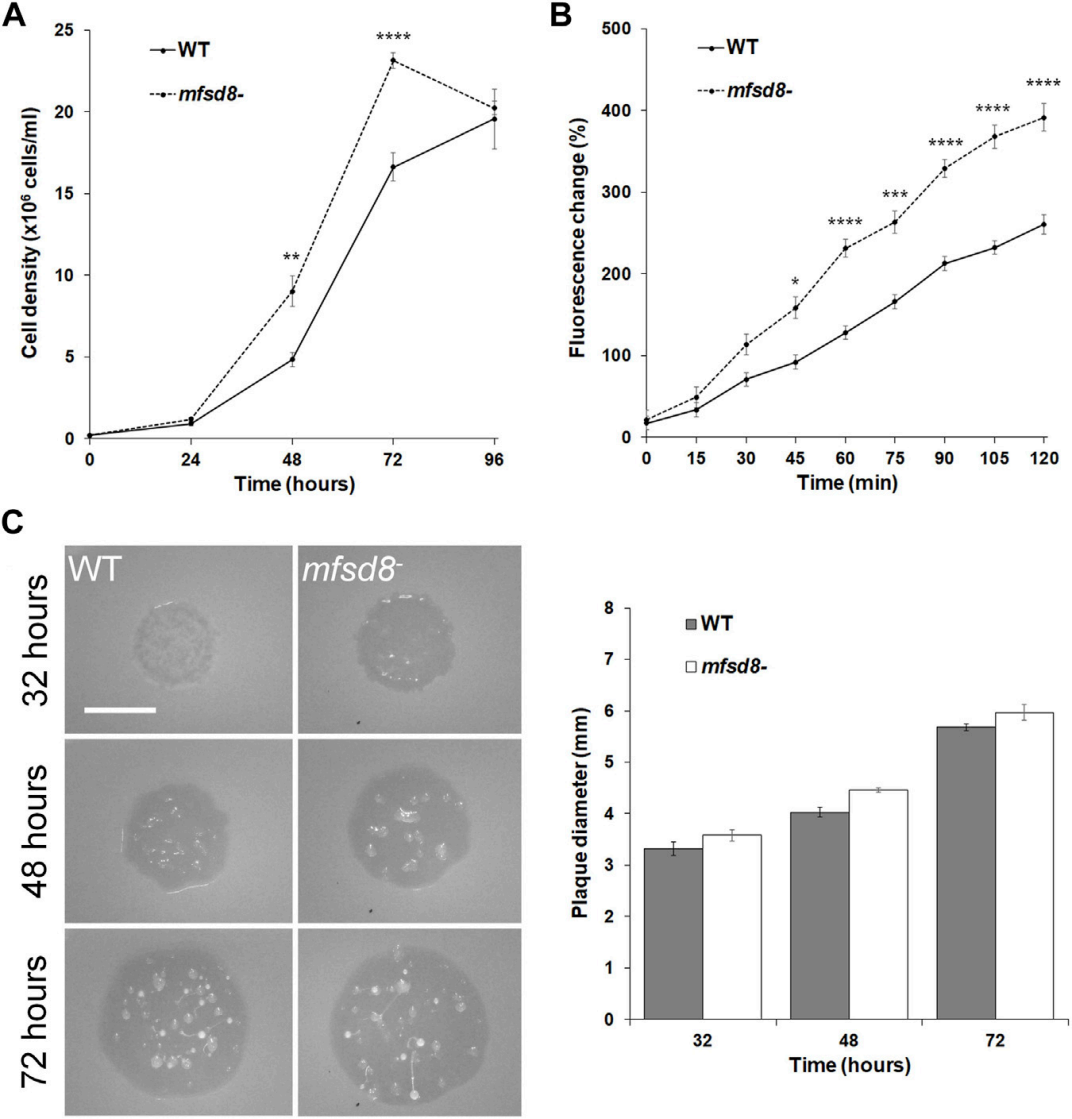
Effect of *mfsd8*-deficiency on cell proliferation, pinocytosis, and plaque expansion. **(A)** Effect of *mfsd8*-deficiency on cell proliferation. WT and *mfsd8* ^−^ cells were grown axenically in HL5 medium. Cell densities were measured every 24 h over a 96-h period. Data presented as mean concentration (×10^6^ cells/ml) ± SEM (*n* = 5). Statistical significance was assessed using two-way ANOVA followed by Bonferroni post-hoc analysis. **p < 0.05 and ****p < 0.0001 vs. WT at the indicated time points. **(B)** Effect of *mfsd8*-deficiency on pinocytosis. WT and *mfsd8* ^−^ cells were incubated in HL5 containing FITC-dextran for 120 min. Cells were collected, washed, and lysed every 15 min. The fluorescence of the lysates was then measured using a plate reader. The data were corrected for background signals and expressed as the mean fluorescence change (%) relative to the 0-min time point. Data presented as mean fluorescence change (%) ± SEM (*n* = 3). Statistical significance was assessed using two-way ANOVA followed by Bonferroni post-hoc analysis. *p < 0.05, ***p < 0.001, and ****p < 0.0001 vs. WT at the indicated time points. **(C)** Effect of *mfsd8*-deficiency on plaque expansion on bacterial lawns. WT and *mfsd8* ^−^ cells grown axenically in HL5 were harvested, washed with KK2 buffer, and deposited onto lawns of *K. aerogenes*. Plaques were imaged at the indicated time points and their diameters were quantified using Fiji/ImageJ. Scale bar = 20 μm. Data presented as mean plaque diameter (mm) ± SEM (*n* = 4).

When cultured in liquid growth medium, *mfsd8* ^*−*^ cells proliferated at a significantly increased rate compared to WT cells (Figure 1A). Since *D. discoideum* cells ingest extracellular liquid nutrients through macropinocytosis, we assessed whether the rate of pinocytosis was affected by *mfsd8*-deficiency (Hacker et al., 1997). Cells were incubated in liquid growth medium containing FITC-dextran for a 120-min period and the amount of intracellular fluorescence was measured every 15 min. Like proliferation, the rate of FITC-dextran accumulation was significantly increased in *mfsd8* ^*−*^ cells relative to WT cells (Figure 1B).

Since loss of *mfsd8* impacted cell proliferation and the accumulation of FITC-dextran in liquid growth medium, we tested whether *mfsd8* ^*−*^ cells would display the same phenotype when grown on bacteria lawns. Cell lines were plated on lawns of *K. aerogenes* and plaque formation was monitored. In general, we observed that *mfsd8* ^*−*^ cells formed plaques earlier than WT cells and the plaques were larger than WT plaques at all time points examined (Figure 1C). Of note, 32 h after depositing cells onto lawns, *mfsd8* ^*−*^ plaques were translucent, while WT plaques were semi-translucent, indicating that *mfsd8* ^*−*^ cells cleared bacterial lawns earlier than WT cells. When feeding on bacterial lawns, *D. discoideum* amoebae chemotactically respond to folic acid that is secreted by bacteria (Pan et al., 1972). However, we observed no significant effect of *mfsd8*-deficiency on folic acid-mediated chemotaxis (Supplementary Figure S1). Together, these results suggest that Mfsd8 regulates cell proliferation, pinocytosis, and growth on bacterial lawns in *D. discoideum*.

### Loss of *mfsd8* Increases Cell Size and Inhibits Cytokinesis During Growth

**FIGURE 2:**
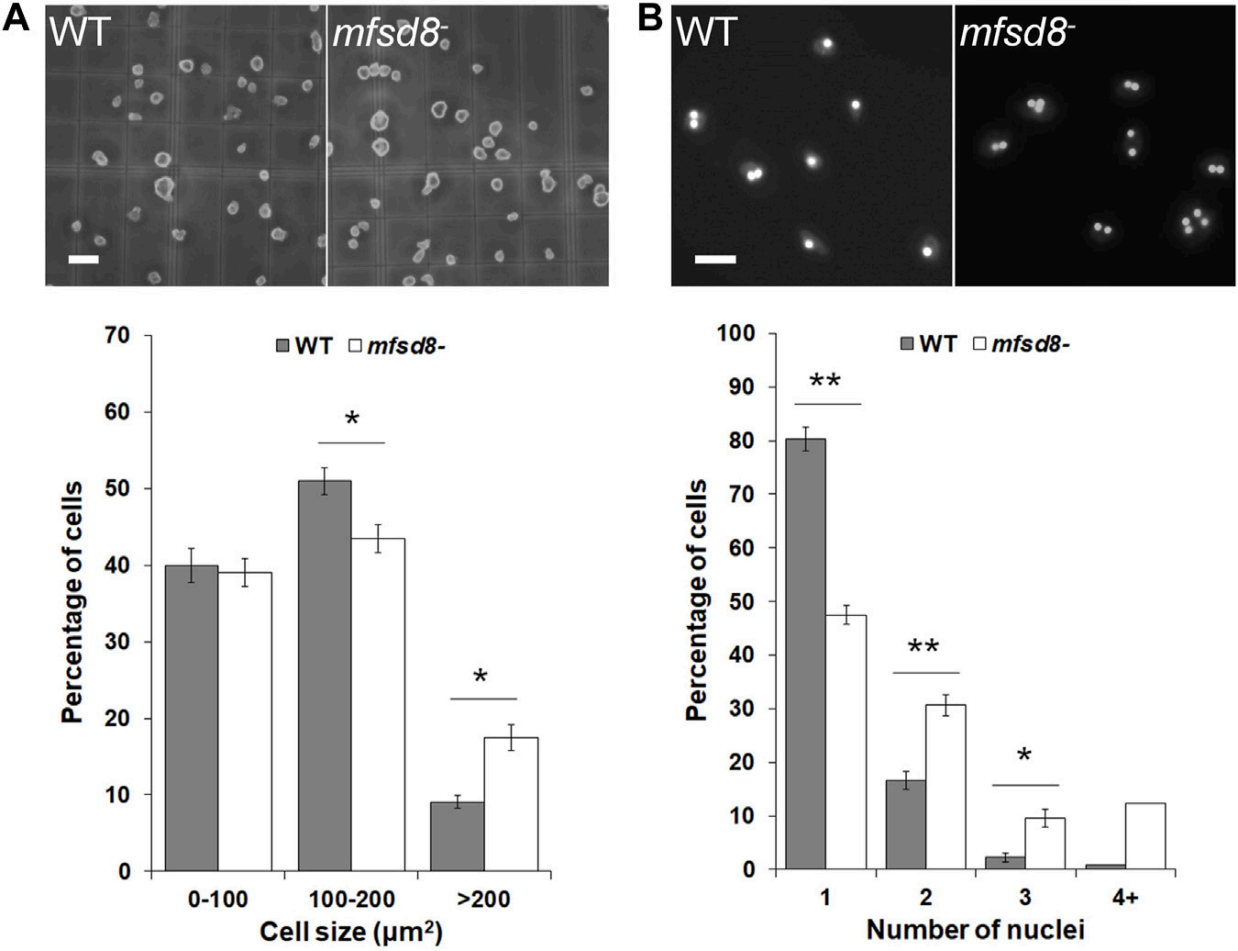
Effect of *mfsd8*-deficiency on cell size and cytokinesis. **(A)** Effect of *mfsd8*-deficiency on cell size. WT and *mfsd8* ^−^ cells grown axenically in HL5 were deposited onto a hemocytometer and imaged. Scale bar = 20 μm. The area of each individual cell was quantified using Fiji/ImageJ. Data was placed into different cell area bins and expressed as a mean percentage of total cells analyzed ± SEM (*n* = 5). Statistical significance was assessed using two-way ANOVA followed by Bonferroni post-hoc analysis. *p < 0.05 vs. WT. **(B)** Effect of *mfsd8*-deficiency on cytokinesis. WT and *mfsd8* ^−^ cells grown axenically in HL5 were collected and deposited onto coverslips placed inside separate wells of a 12-well dish. Cells on coverslips were then submerged in low-fluorescence HL5 overnight at 21°C. The following day, cells were fixed in −80°C methanol mounted onto slides with Prolong Gold Anti-Fade Reagent containing DAPI. Scale bar = 20 μm. The number of nuclei within each cell was scored. For each experiment, 10 random images, each containing at least 10 cells, were obtained from each coverslip. Data presented as mean percentage of total cells analyzed ±SEM (*n* = 7). Statistical significance was assessed using two-way ANOVA followed by Bonferroni post-hoc analysis. *p < 0.05 and **p < 0.01 vs. WT.

When examining the proliferation of *mfsd8* ^*−*^ cells, we observed that *mfsd8* ^*−*^ cells appeared larger than WT cells. Indeed, when we quantified the area of cells cultured in liquid growth medium, we found that *mfsd8* ^*−*^ cultures contained a significantly higher proportion of cells > 200 μm^2^ compared to WT cultures, and a correlated lower proportion of cells 100–200 μm^2^ (Figure 2A).

When cultured in liquid growth medium, the majority of *D. discoideum* cells are mononucleated. However, it is well established that axenically grown cultures of *D. discoideum* can also contain polynucleated cells, which form due to defects in cytokinesis and tend to be larger than mononucleated cells (Waddell et al., 1987). Based on these findings, we examined the proportions of mononucleated and polynucleated cells in *mfsd8* ^*−*^ cultures. We observed that loss of *mfsd8* significantly decreased the proportion of mononucleated cells in culture and increased the proportion of polynucleated cells (Figure 2B). Together, these findings showed that *mfsd8*-deficiency increases cell size and reduces cytokinesis during the growth phase of the *D. discoideum* life cycle.

### Loss of *mfsd8* Alters the Levels of Autocrine Proliferation Repressor During Growth

**FIGURE 3:**
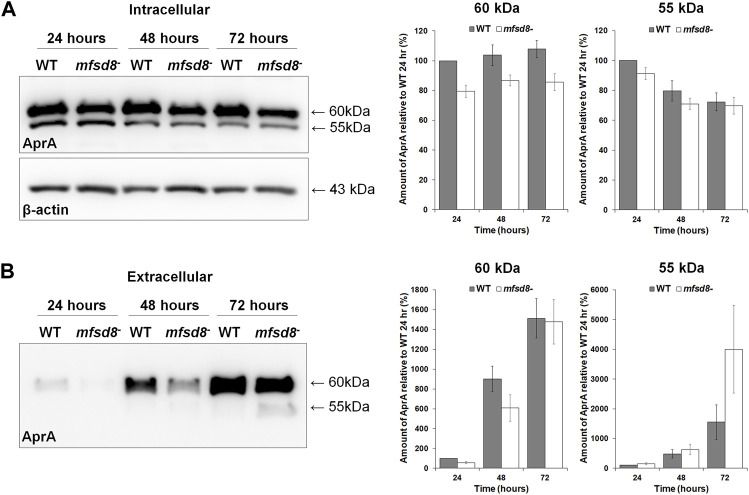
Effect of *mfsd8*-deficiency on the intracellular and extracellular levels of AprA during growth. WT and *mfsd8* ^−^ cells were grown in HL5, harvested after 24, 48, and 72 h of growth, and lysed. Conditioned media (CM) was also harvested at each time point. **(A)** Whole cell lysates (15 µg) were separated by SDS-PAGE and analyzed by western blotting with anti-AprA and anti-β-actin (loading control). Molecular weight markers (in kDa) are shown to the right of each blot. Protein bands were quantified using Fiji/ImageJ. The amounts of AprA were normalized against the amounts of β-actin. Data presented as mean amount of AprA relative to WT 24 h (%) ± SEM (*n* = 7). **(B)** Samples of CM were harvested at each time point and standardized against cell number. Equal volumes of CM (15 μl) were separated by SDS-PAGE and analyzed by western blotting with anti-AprA. Molecular weight markers (in kDa) are shown to the right of each blot. Protein bands were quantified using Fiji/ImageJ. Data presented as mean amount of AprA relative to WT 24 h (%) ± SEM (*n* = 7).

To gain insight into the possible mechanisms underlying the increased proliferation of *mfsd8* ^*−*^ cells, the intracellular and extracellular levels of AprA were examined. AprA functions extracellularly to repress cell proliferation in *D. discoideum* and has previously been shown to be aberrantly secreted by *cln3* ^*−*^ cells, which like *mfsd8* ^*−*^ cells, also display increased rates of proliferation (Brock and Gomer, 2005; Huber et al., 2014). WC lysates and CM from WT and *mfsd8* ^*−*^ cells were collected after 24, 48, and 72 h of axenic growth (Figure 1A). When analyzed by western blotting, anti-AprA detected two protein bands; one at 60 kDa and the other at 55 kDa, which is consistent with the banding pattern reported in previous studies (Brock and Gomer, 2005; Huber et al., 2014) (Figure 3). Intracellularly, the amounts of 60 kDa AprA remained constant during all stages of axenic growth for both WT and *mfsd8* ^*−*^ cultures (Figure 3A). However, the amounts of 60 kDa AprA in *mfsd8* ^*−*^ cells were lower at all time points analyzed compared to WT cells. The amounts of intracellular 55 kDa AprA decreased in WT and *mfsd8* ^*−*^ cells during axenic growth but there were no differences between cell lines. In samples of CM, loss of *mfsd8* reduced the extracellular amount of 60 kDa AprA after 24 and 48 h of axenic growth, compared to WT cells, but there was no effect at the 72-h time point (Figure 3B). In addition, CM collected from *mfsd8* ^*−*^ cells after 72 h of axenic growth contained more 55 kDa AprA than CM collected from WT cells. Blots containing samples of CM were also probed with anti-β-actin to validate that CM did not contain intracellular proteins due to cell lysis (data not shown). Since AprA functions as a proliferation repressor, these findings suggest that altered levels of intracellular and extracellular AprA may have contributed to the increased rate of proliferation of *mfsd8* ^*−*^ cells.

### *mfsd8*-Deficiency Increases the Intracellular Activities of Several Lysosomal Enzymes During Growth

**FIGURE 4:**
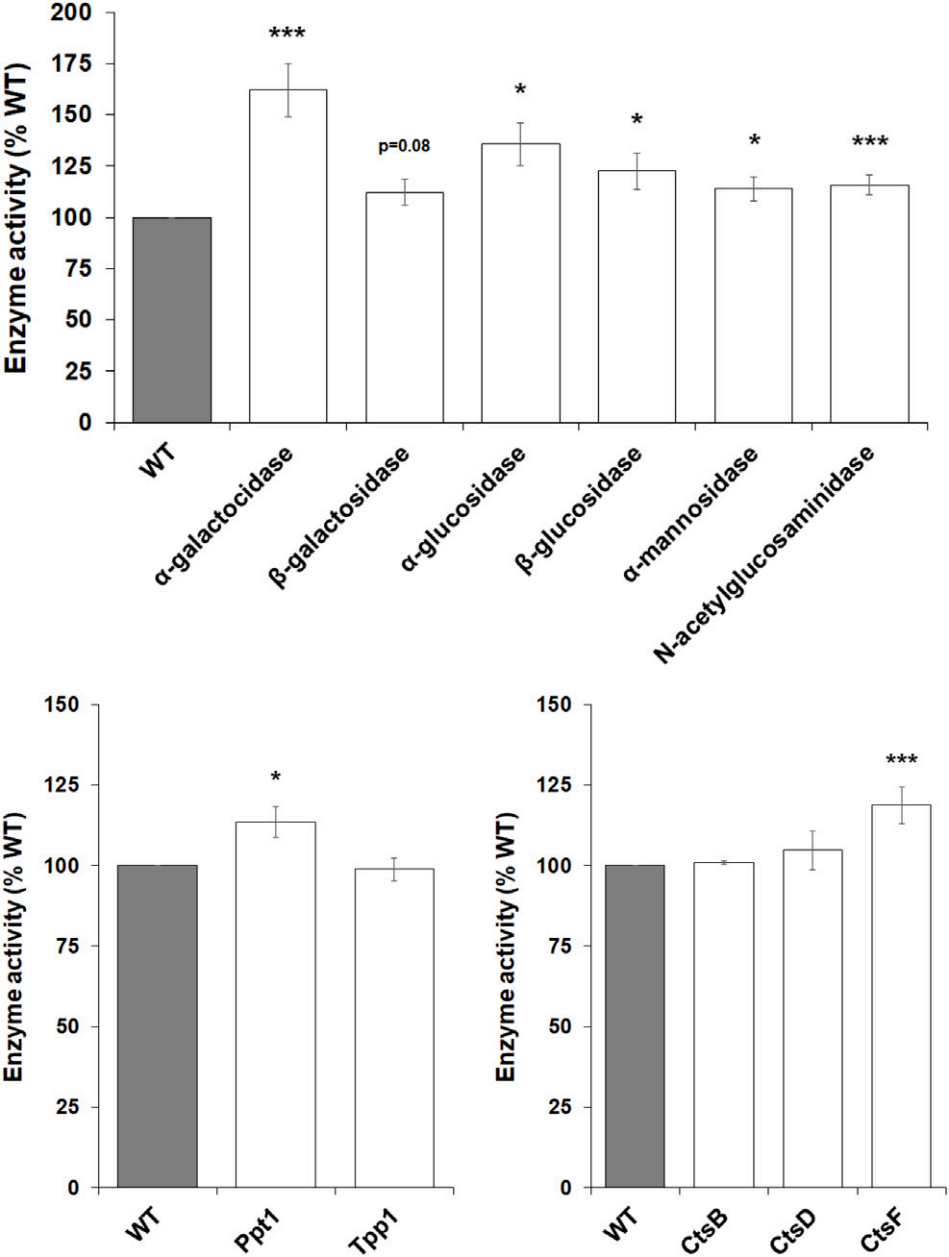
Effect of *mfsd8*-deficiency on enzyme activity during growth. WT and *mfsd8* ^−^ cells grown axenically in HL5 were collected and lysed. The activities of α-galactosidase, β-galactosidase, α-glucosidase, β-glucosidase, α-mannosidase, N-acetylglucosaminidase, palmitoyl-protein thioesterase 1 (Ppt1), tripeptidyl peptidase 1 (Tpp1), cathepsin B (CtsB), cathepsin D (CtsD), and cathepsin F (CtsF) were assessed as described in the Materials and Methods. Data presented as mean enzyme activity (% WT) ± SEM (*n* > 4). Statistical significance was determined using the one-sample *t*-test (mean, 100; two-tailed) vs. WT. *p < 0.05 and ***p < 0.001 vs. WT.

Lysosomal enzymes play an essential role in degrading material internalized by amoebae during the growth phase of the *D. discoideum* life cycle to provide cells with nutrients (Ashworth and Quance, 1972). Interestingly, in mice, loss of *Mfsd8* has been shown to affect the levels of several lysosomal enzymes (Danyukova et al., 2018). Thus, we were interested in assessing lysosomal enzyme activity in *mfsd8* ^*−*^ cells during growth. Loss of *mfsd8* significantly increased the intracellular activities of α-galactosidase, α-glucosidase, β-glucosidase, α-mannosidase, and N-acetylglucosaminidase (Figure 4). The activity of β-galactosidase was also slightly elevated but not statistically significant (*p* = 0.08). *mfsd8*-deficiency also elevated the activities of Ppt1 and CtsF. In humans, mutations in *PPT1* and *CTSF* cause the CLN1 and CLN13 subtypes of NCL, respectively (Mole and Cotman, 2015). Finally, there was no effect of *mfsd8* loss on the activities of Tpp1, CtsB, or CtsD. In humans, mutations in *TPP1* and *CTSD* cause the CLN2 and CLN10 subtypes of NCL, respectively, and CTSB has been identified as a potential biomarker for CLN6 disease (Huber, 2021). Together, these data suggest that *mfsd8* ^*−*^ cells increase the activities of some, but not all, lysosomal enzymes to support their increased rates of proliferation and pinocytosis.

### Loss of *mfsd8* Delays Aggregation During the Early Stages of Multicellular Development

**FIGURE 5:**
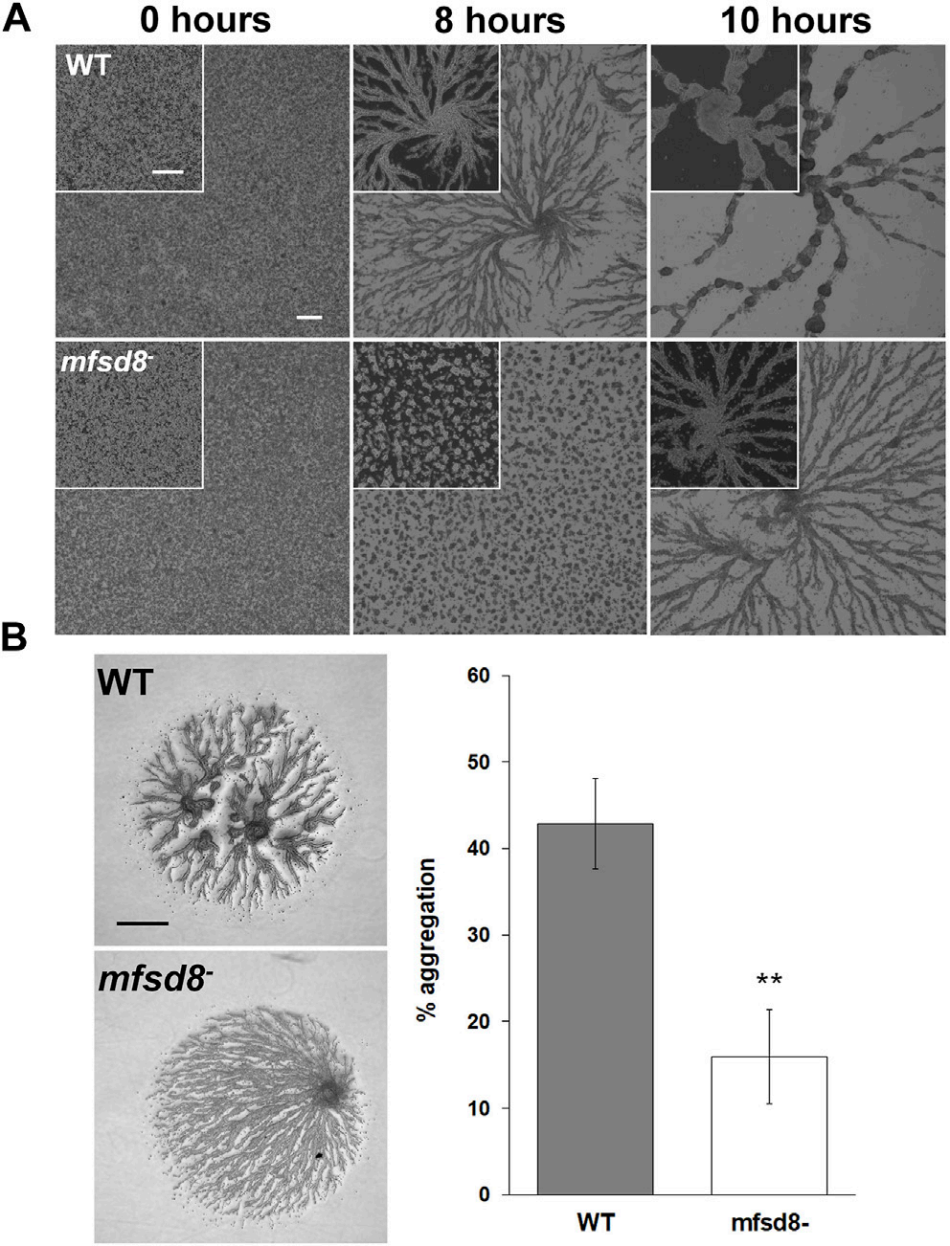
Effect of *mfsd8*-deficiency on aggregation. **(A)** WT and *mfsd8* ^−^ cells grown axenically in HL5 were submerged in KK2 buffer and imaged at the indicated time points. Scale bars = 250 μm. Images are representative of six independent experiments. **(B)** WT and *mfsd8* ^−^ cells grown axenically in HL5 were harvested, washed with KK2 buffer, and deposited onto 0.5% agar/KK2. Images were taken once cells were deposited and after 5 h. Scale bar = 250 μm. The amount of aggregation of each cell line was quantified by measuring the area of the cell spot that remained after 5 h of starvation, subtracting it from the initial area, and then expressing the difference as a percentage of the area of the cell spot at 0 h. Data presented as mean % aggregation ± SEM (*n* = 9). Statistical significance was assessed using the Kruskal-Wallis test followed by the Dunn’s multiple comparisons test. **p < 0.01.

Based on the increased expression of *mfsd8* during the early stages of development (Rot et al., 2009), we suspected that loss of *mfsd8* would affect cellular processes during this stage of the life cycle. Therefore, we performed two assays to examine the impact of *mfsd8*-deficiency on aggregation. In the first assay, cells adhered to Petri dishes were submerged in KK2 buffer to initiate the developmental program. After 8 h, there was a noticeable delay in the aggregation of *mfsd8* ^*−*^ cells compared to WT cells (Figure 5A). A delay in *mfsd8* ^*−*^ aggregation was also observed when cells were deposited on KK2-buffered agar (Figure 5B). In *D. discoideum*, mound formation occurs through the chemotactic aggregation of cells towards 3′,5′-cyclic adenosine monophosphate (cAMP) (Konijn et al., 1967). However, we did not observe a significant effect of *mfsd8*-deficiency on cAMP-mediated chemotaxis (Supplementary Figure S1).

**FIGURE 6:**
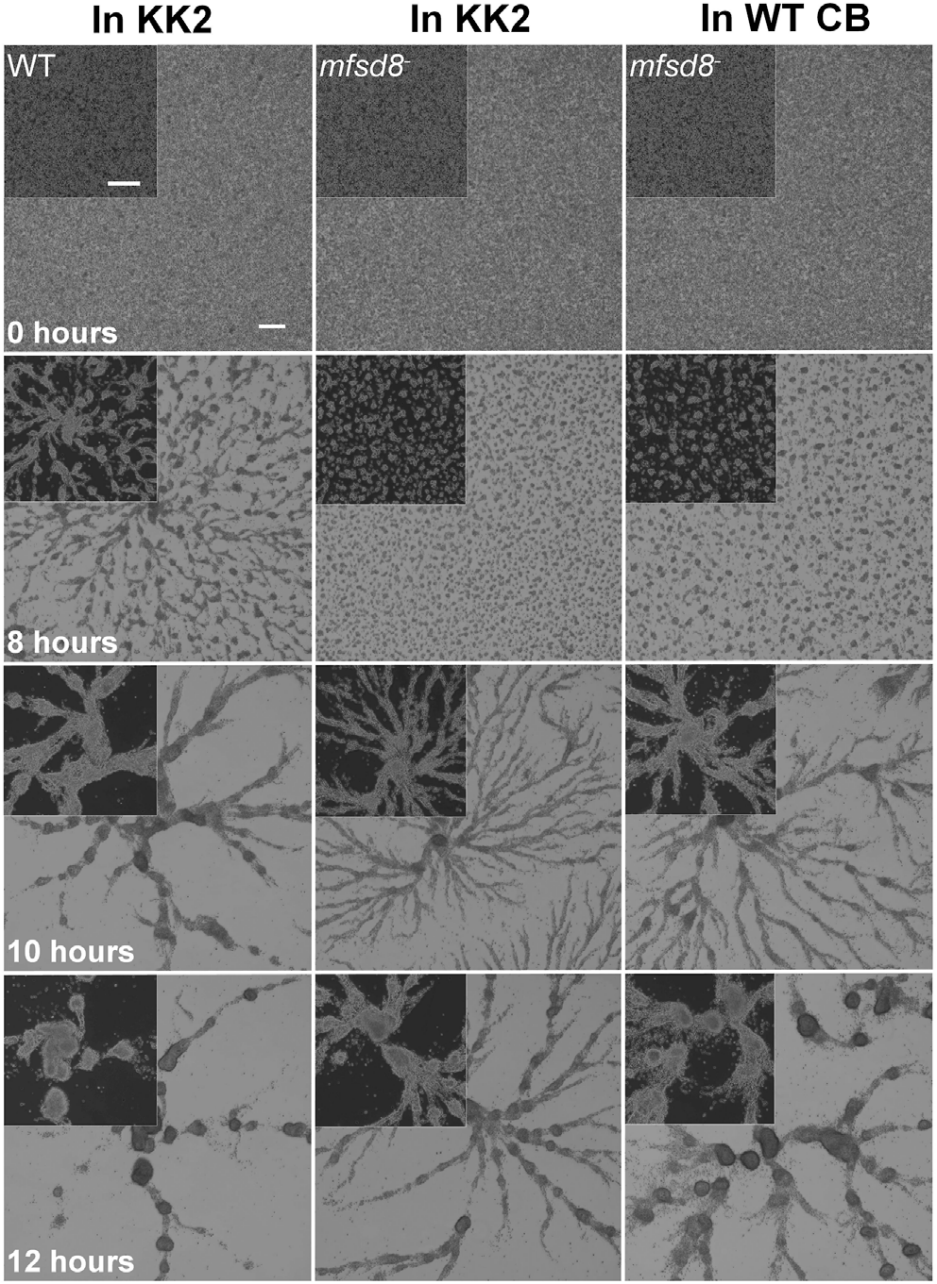
Effect of conditioned buffer on the aggregation of *mfsd8* ^−^ cells. WT cells were submerged in KK2 buffer for 2 h, after which time, the conditioned buffer (CB) of starving WT cells was collected and added to *mfsd8* ^−^ cells at the onset of starvation. Images are representative of nine independent experiments. Scale bar = 250 μm.

**FIGURE 7:**
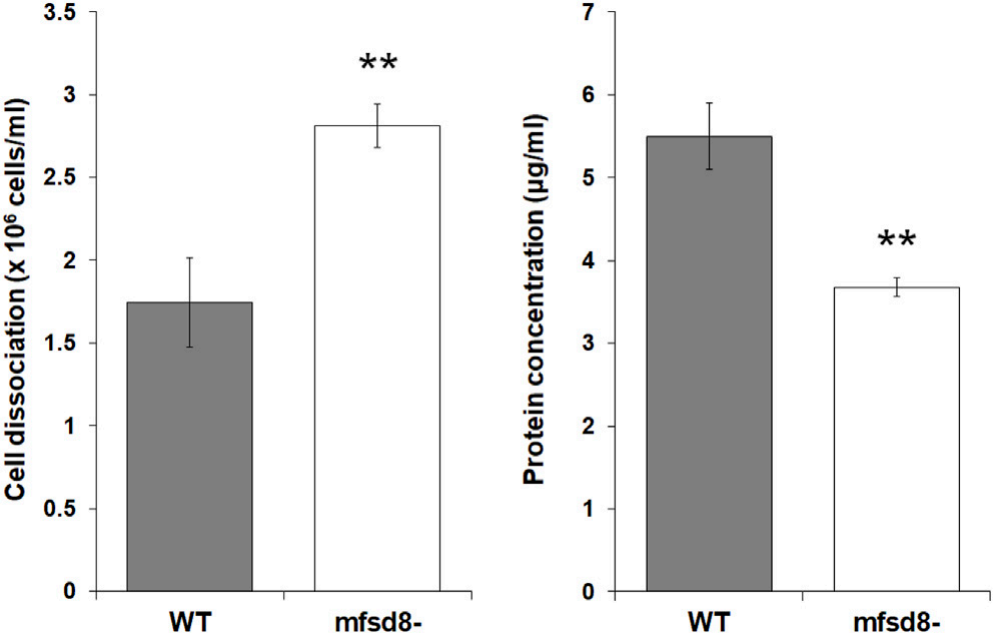
Effect of *mfsd8*-deficiency on cell-substrate adhesion. WT and *mfsd8* ^−^ cells were submerged in KK2 buffer for 4 h, after which time they were shaken at 150 rpm for 30 min. Samples of conditioned buffer were collected to measure cell dissociation, while cells remaining on the dish were lysed to assess protein concentration. Data presented as mean cell dissociation (×10^6^ cells/ml) and protein concentration (μg/ml) ± SEM (*n* = 9). Statistical significance was assessed using one-way ANOVA followed by Bonferroni post-hoc analysis. **p < 0.01 vs. WT.

A previous study showed that *mfsd8*-deficiency alters the secretion of two other CLN protein homologs in *D. discoideum*, Cln5 and CtsD, during the early stages of development (Huber et al., 2020b). Thus, we performed a CB swap experiment to examine the possible role of altered secretion in the delayed aggregation of *mfsd8* ^*−*^ cells. For this assay, we starved WT cells for 2 h and then collected the CB. In nine independent experiments, we observed that incubating *mfsd8* ^*−*^ cells in CB collected from WT cells partially restored the delayed aggregation of *mfsd8* ^*−*^ cells relative to WT cells (Figure 6). We also examined the effect of *mfsd8*-deficiency on cell-substrate adhesion, which plays an essential role in aggregation (Tarantola et al., 2014). During the early stages of development, loss of *mfsd8* caused more cells to de-adhere from Petri dishes relative to WT cells (Figure 7). Combined, these data suggest that reduced cell-substrate adhesion along with aberrant protein secretion likely contribute to the delayed aggregation of *mfsd8* ^*−*^ cells during the early stages of *D. discoideum* development.

### *mfsd8*-Deficiency Affects the Intracellular and Extracellular Amounts of Proteins Involved in Aggregation and Cell Adhesion

**FIGURE 8:**
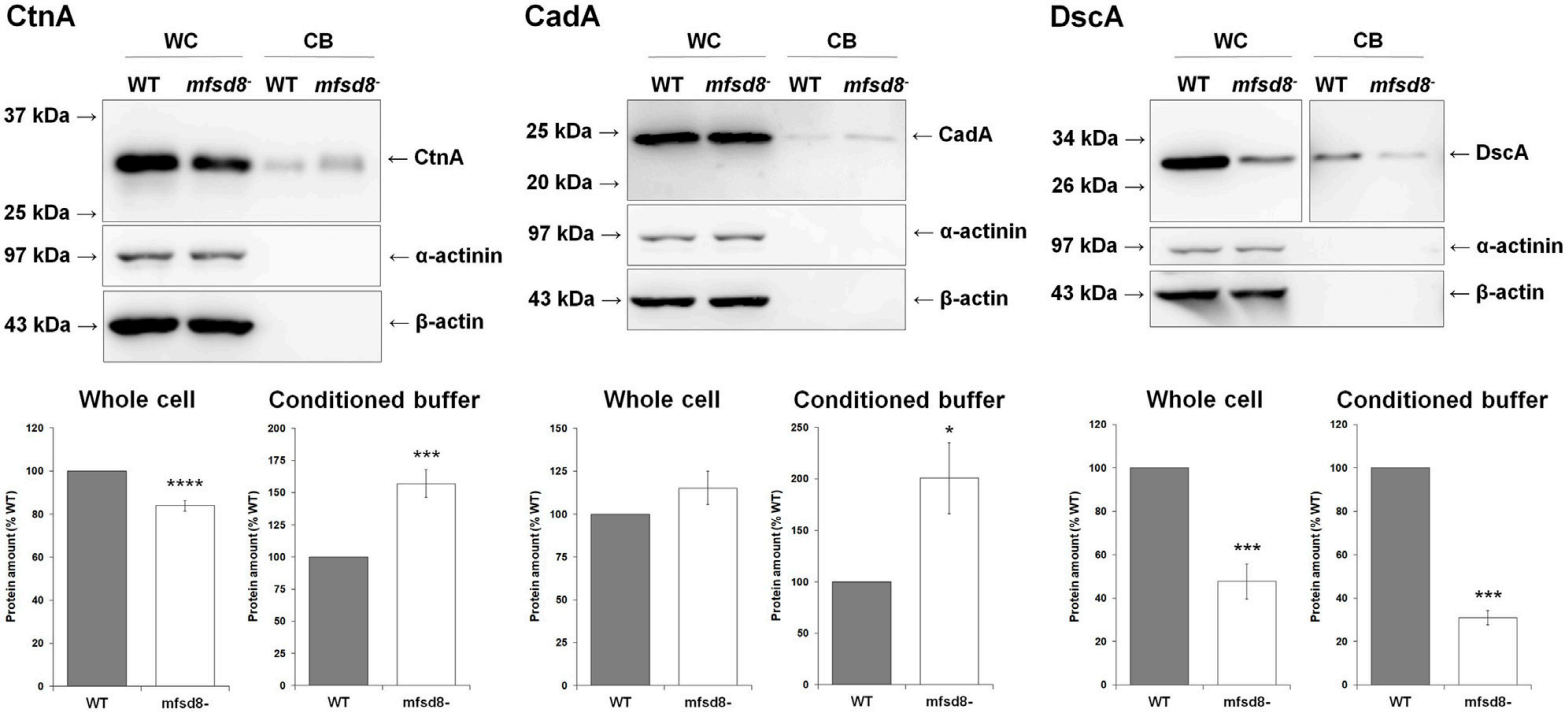
Effect of *mfsd8*-deficiency on the intracellular and extracellular levels of CtnA, CadA, and DscA. Cells grown axenically in HL5 were starved for 4 h in KK2 buffer, after which time whole cell (WC) lysates and conditioned buffer (CB) were collected. WC lysates (15 μg) and concentrated CB (0.25 μg) were separated by SDS-PAGE and analyzed by western blotting with anti-CtnA, anti-CadA, anti-DscA, anti-α-actinin (loading and fractionation control), and anti-β-actin (loading and fractionation control). Protein bands from WC lysates were standardized against the levels of α-actinin and β-actin. Data presented as mean protein amount (% WT) ± SEM (*n* = 8). Statistical significance was assessed using the one sample *t*-test. *p < 0.05, ***p < 0.001, and ****p < 0.0001 vs. WT.

To gain further insight into the cellular mechanisms affected by *mfsd8*-deficiency, we starved WT and *mfsd8* ^*−*^ cells for 4 h and analyzed the intracellular and extracellular amounts of CtnA, CadA, and DscA. CtnA is a component of a secreted 450 kDa protein complex that regulates group size during aggregation by repressing cell-cell adhesion (Roisin-Bouffay et al., 2000). CadA and DscA, which were previously shown to interact with Mfsd8 (Huber et al., 2020b), both play important roles in regulating cell adhesion during the early stages of development (Cano and Pestaña, 1984; Springer et al., 1984; Knecht et al., 1987; Brar and Siu, 1993). In addition, loss of *dscA* impairs cell-substrate adhesion during aggregation (Springer et al., 1984; Bastounis et al., 2016). In this study, loss of *mfsd8* significantly reduced the intracellular level of CtnA but increased the amount of the protein in CB, suggesting that secretion of CtnA was increased due to *mfsd8*-deficiency (Figure 8). Loss of *mfsd8* had no effect on the amount of CadA inside cells but significantly increased its level extracellularly. Finally, loss of *mfsd8* reduced the intracellular and extracellular levels of DscA. Combined, these data indicate that *mfsd8*-deficiency affects the intracellular and extracellular amounts of proteins involved in aggregation and cell adhesion, which could explain the delayed aggregation and reduced adhesion of *mfsd8* ^*−*^ cells during the early stages of development.

### Loss of *mfsd8* ^*−*^ Alters Lysosomal Enzyme Activities During the Early Stages of Development

**FIGURE 9:**
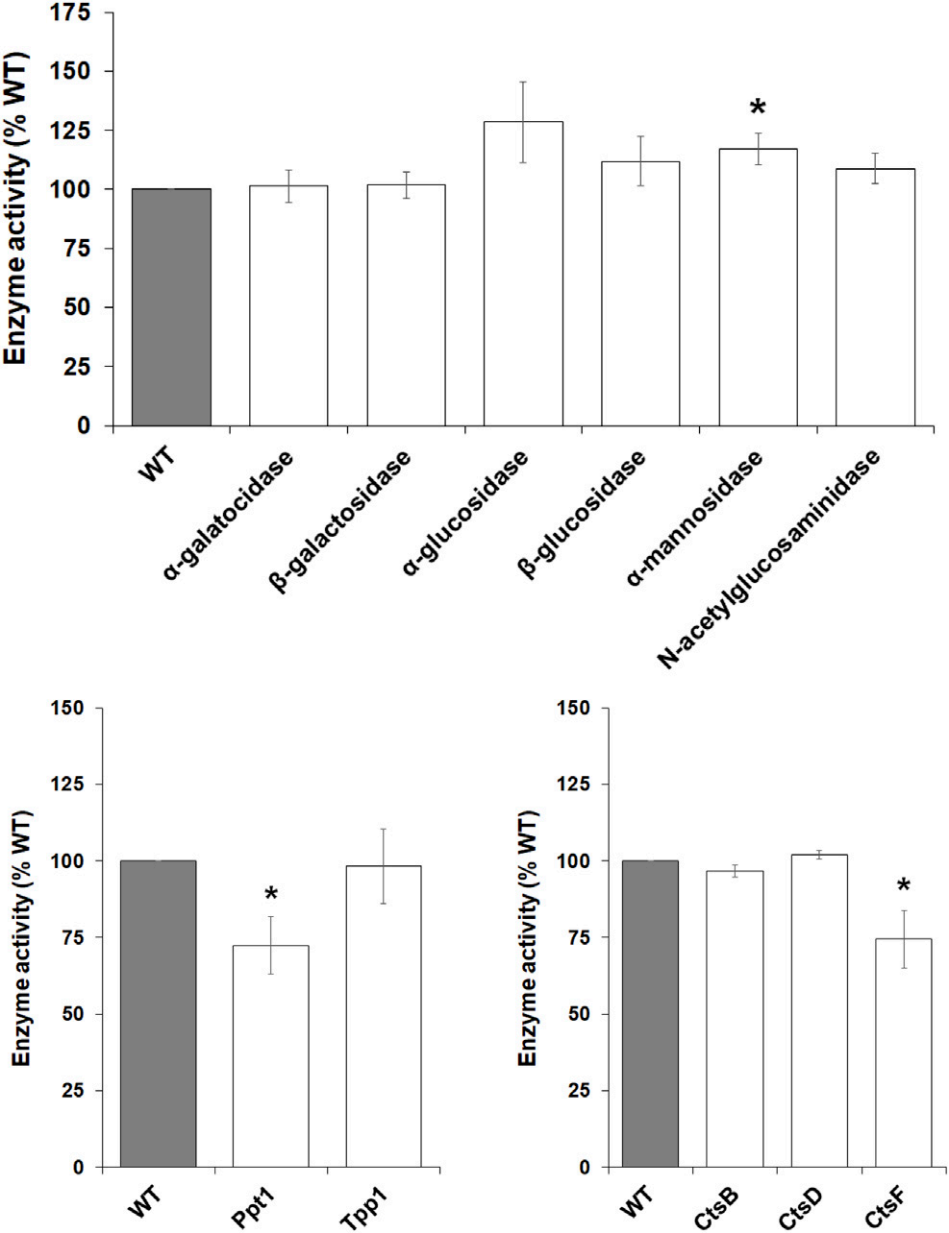
Effect of *mfsd8*-deficiency on enzyme activity during starvation. WT and *mfsd8* ^−^ cells grown axenically in HL5 were starved in KK2 buffer for 4 h, after which time cells were lysed. The activities of α-galactosidase, β-galactosidase, α-glucosidase, β-glucosidase, α-mannosidase, N-acetylglucosaminidase, palmitoyl-protein thioesterase 1 (Ppt1), tripeptidyl peptidase 1 (Tpp1), cathepsin B (CtsB), cathepsin D (CtsD), and cathepsin F (CtsF) were assessed as described in the Materials and Methods. Data presented as mean enzyme activity (% WT) ± SEM (*n* > 4). Statistical significance was determined using the one-sample *t*-test (mean, 100; two-tailed) vs. WT. *p < 0.05 and ***p < 0.001 vs. WT.

During multicellular development, *D. discoideum* amoebae rely on autophagy and the actions of several lysosomal enzymes to provide cells with energy and building blocks required for fueling aggregation and fruiting body formation (Loomis, 1969; Loomis, 1970; Dimond et al., 1973; Kilpatrick and Stirling, 1976; Knecht et al., 1985; Otto et al., 2003; Kiel, 2010). As noted above, previous work in mice showed that loss of *Mfsd8* affects the levels of several lysosomal enzymes (Danyukova et al., 2018). Thus, we assessed the effect of *mfsd8*-deficiency on lysosomal enzyme activity during the early stages of *D. discoideum* development. After 4 h of starvation, we observed increased intracellular activity of α-mannosidase and decreased activity of Ppt1 and CtsF (Figure 9). There was no significant effect of *mfsd8*-deficiency on the activities of α-galactosidase, β-galactosidase, α-glucosidase, β-glucosidase, N-acetylglucosaminidase, Tpp1, CtsB, or CtsD. Combined, these findings show that loss of *mfsd8* alters the activities of some, but not all, lysosomal enzymes during aggregation.

## Discussion

**FIGURE 10:**
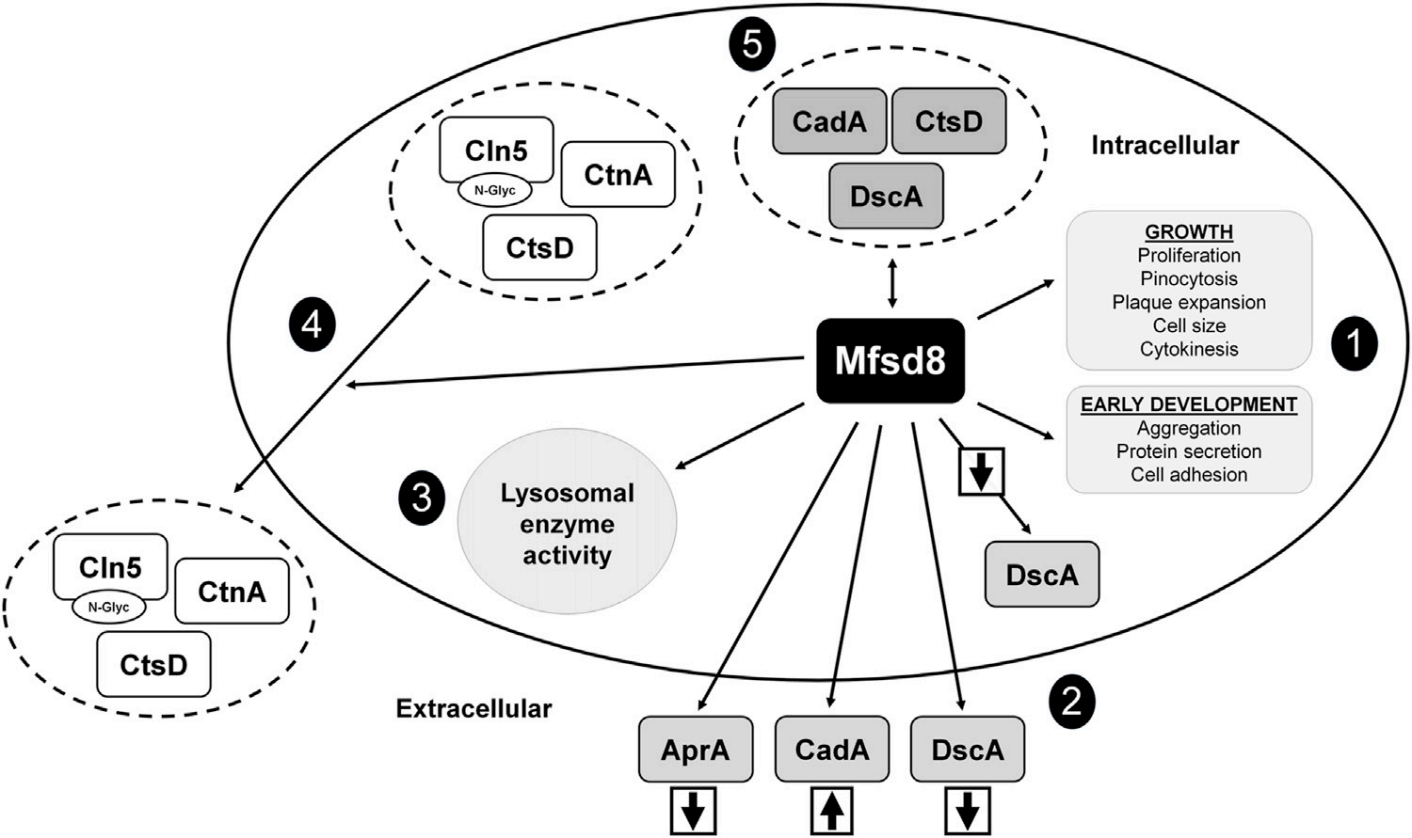
Current model depicting the functions of Mfsd8 in *D. discoideum*. (1) Loss of *mfsd8* affects cell proliferation, pinocytosis, plaque expansion, cell size, and cytokinesis during growth, and aggregation, protein secretion, and cell adhesion during the early stages of development. (2) Loss of *mfsd8* reduces the intracellular and extracellular amounts of DscA during the early stages of development and increases the extracellular amount of CadA. Loss of *mfsd8* decreases the extracellular amount of AprA during growth. (3) Loss of *mfsd8* affects lysosomal enzyme activity during growth and the early stages of development. (4) Mfsd8 regulates the secretion of Cln5, CtsD, and CtnA during the early stages of development. (5) Mfsd8 interacts with CadA, CtsD, and DscA during growth and the early stages of development.

In this study, we examined the cellular roles of Mfsd8 during the growth and early development of *D. discoideum* (Figure 10). During growth, *mfsd8*-deficiency enhanced cell proliferation, FITC-dextran accumulation, and growth on bacterial lawns. The increased proliferation of *mfsd8* ^*−*^ cells correlated with altered levels of the proliferation repressor AprA and increased activity of several lysosomal enzymes. We also showed that Mfsd8 functions during the early stages of development to regulate cell adhesion, protein secretion, lysosomal enzyme activity, and aggregation. Together, this study provides new insights into the multifaceted roles of MFSD8 in the eukaryotic cell.

The enhanced proliferation and accumulation of FITC-dextran observed in *mfsd8* ^*−*^ cells suggests that the increased proliferation was at least partly due to increased nutrient uptake. When grown on bacterial lawns, *mfsd8* ^*−*^ cells form plaques earlier than WT cells, further supporting their increased rate of growth. However, we showed that loss of *mfsd8* has no effect on folic acid-mediated chemotaxis, which drives bacterial acquisition during feeding (Pan et al., 1972), suggesting that Mfsd8 influences mechanisms other than folic acid signalling during bacterial uptake.

Mfsd8 localizes to the macropinocytosis pathway in *D. discoideum* (Journet et al., 2012). In addition, previous work has shown that Mfsd8 interacts with proteins that negatively regulate endocytosis such as the Ras GTPase RapA and the nucleoside diphosphate kinase NdkC-1 (Seastone et al., 1999; Annesley et al., 2011). As a result, the loss of *mfsd8* could have impacted the ability of these proteins to limit the rate of endocytosis in *mfsd8* ^*−*^ cells. Mfsd8-interactors also include transport proteins as well as proteins that localize to endocytic vesicles and the cytoskeleton (e.g., actin-10, myosin-2 heavy chain, tubulin alpha chain, and V-ATPase subunit B) (Huber et al., 2020b). Since endocytic processes are highly dependent on cytoskeletal elements for membrane invagination and vesicle transport (Kumari et al., 2010; Mooren et al., 2012), these observations provide insight into the mechanisms underlying the role of Mfsd8 in pinocytosis.

Loss of *mfsd8* increases cell size, which like proliferation, could be explained by increased liquid nutrient uptake. However, we also observed that *mfsd8*-deficiency reduces cytokinesis, which is consistent with previous work in *D. discoideum* that correlated reduced cytokinesis and enhanced pinocytosis to increased cell size (Adachi, 2001; Winckler et al., 2001; Lim et al., 2005). In addition, enhanced proliferation and reduced cytokinesis have been observed in other *D. discoideum* mutants (Brock and Gomer, 2005; Bakthavatsalam et al., 2008; Huber et al., 2014; Mathavarajah et al., 2018). In this study, we observed reduced cytokinesis of *mfsd8* ^*−*^ cells submerged in growth medium on dishes. We suspect the cytokinesis defect could be due to aberrant adhesion, which is consistent with our observations of reduced adhesion of *mfsd8* ^*−*^ cells and previous work in *D. discoideum* linking aberrant cytokinesis to defects in adhesion (Nagasaki et al., 2009; Tsujioka et al., 2012). Finally, Mfsd8 was previously shown to interact with several proteins involved in cytokinesis, including actin-10, RapA, myosin-2 heavy chain, and Ras-like protein RasG (Tuxworth et al., 1997; Huber et al., 2020b). Together, these findings support a role for Mfsd8 in cytokinesis.

In *D. discoideum,* AprA is a secreted factor that represses cell proliferation and shares structural and functional similarity with human dipeptidyl peptidase 4 (Brock and Gomer, 2005; Herlihy et al., 2013; Herlihy et al., 2017). In addition to repressing cell proliferation, AprA also coordinates cytokinesis following mitosis and helps to reduce the formation of multinucleated cells (Brock and Gomer, 2005). In this study, extracellular AprA accumulated in parallel with cell density, which aligns with observations in previous studies (Brock and Gomer, 2005; Huber et al., 2014). However, loss of *mfsd8* reduced the intracellular and extracellular levels of 60 kDa AprA suggesting that Mfsd8 may regulate proliferation by modulating both the synthesis and secretion of AprA. Contrary to the levels of 60 kDa AprA, loss of *mfsd8* dramatically increased the extracellular levels of 55 kDa AprA as cells approached the stationary phase of axenic growth. While the identity of the 55 kDa band is not known, it has been proposed to be a AprA cleavage product (Huber et al., 2014). Therefore, its increased presence in *mfsd8* ^*−*^ CM during the later stages of axenic growth could be due to AprA degradation once cells reach the stationary phase of growth, which is consistent with our observation of *mfsd8* ^*−*^ cells reaching stationary phase earlier than WT cells. Combined, these observations suggest that loss of *mfsd8* affects the synthesis and secretion of AprA, which may have played a role in the aberrant proliferation and cytokinesis observed in *mfsd8* ^*−*^ cells.

Lysosomes play an essential role in degrading endocytosed material to simple metabolites (Tjelle et al., 1996; Pillay et al., 2002). A recent study using MEFs derived from a *Mfsd8* ^*−/−*^ mouse showed that loss of *Mfsd8* alters the amounts of soluble lysosomal proteins (Danyukova et al., 2018). In addition, our previous work showed that 61% of Mfsd8-interactors during growth have catalytic activity (Huber et al., 2020b). In this study, we observed that loss of *mfsd8* increases the intracellular activities of several lysosomal enzymes, including α-galactosidase, α-glucosidase, β-glucosidase, α-mannosidase, N-acetylglucosaminidase, Ppt1, and CtsF. While these observations suggest that Mfsd8 plays a role in regulating lysosomal enzyme activity, the increased activities of these and potentially other lysosomal enzymes could also be explained by *mfsd8* ^*−*^ cells ingesting material at an increased rate. The increased lysosomal enzyme activity would allow material to be digested at an enhanced rate to prevent its accumulation and/or provide macromolecules required to fuel the increased rate of proliferation. Although one could argue that the increased intracellular fluorescence in *mfsd8* ^*−*^ cells could be due to reduced degradation of the internalized material, this seems unlikely since the increased lysosomal enzyme activity suggests that degradation of internalized material is normal in *mfsd8* ^*−*^ cells. Finally, while loss of *mfsd8* affected CtsF activity, there are several proteins in *D. discoideum* that share sequence similarity with human CTSF (Huber et al., 2020a). Therefore, it is currently not known which CTSF-like protein(s) in *D. discoideum* is/are affected by *mfsd8*-deficiency.

Here, we showed that loss of *mfsd8* delays aggregation but has no effect on cAMP-mediated chemotaxis. In addition, submerging *mfsd8* ^*−*^ cells in CB harvested from starving WT cells partially restored the timing of aggregation to WT levels. These findings support a regulatory role for Mfsd8 in processes involved in aggregation and suggest that Mfsd8 may regulate protein secretion. In a previous study, we showed that *mfsd8*-deficiency alters the secretion of Cln5 and CtsD (Huber et al., 2020b). Here, we showed that loss of *mfsd8* also modulates the secretion of CtnA. However, we did not observe any obvious effects of *mfsd8*-deficiency on global protein secretion during growth or starvation (data not shown). Thus, it seems that Mfsd8 may regulate the secretion of selected proteins *via* an undetermined pathway to regulate aggregation.

*mfsd8*-deficiency also affected cell adhesion during the early stages of development. Consistent with this phenotype, we observed increased extracellular amounts of CadA in *mfsd8* ^*−*^ CB and reduced intracellular and extracellular amounts of DscA. Thus, it appears that altered levels of these two cell adhesion proteins likely contributed to the reduced adhesion of *mfsd8* ^*−*^ cells. This is further supported by work that has reported impaired cell-substrate adhesion for cells lacking *dscA* (Springer et al., 1984; Bastounis et al., 2016). Intriguingly, both CadA and DscA were identified in the Mfsd8-interactome (Huber et al., 2020b). In addition, the increased secretion of CtnA also likely contributed to the reduced adhesion of *mfsd8* ^*−*^ cells, since increased extracellular CtnA has been shown to reduce adhesion (Roisin-Bouffay et al., 2000).

During multicellular development, *D. discoideum* amoebae utilize autophagy and lysosomal enzymes to provide cells with energy required for fruiting body formation (Loomis, 1969; Loomis, 1970; Dimond et al., 1973; Kilpatrick and Stirling, 1976; Knecht et al., 1985; Otto et al., 2003; Kiel, 2010). In this study, we observed increased activity of α-mannosidase in *mfsd8* ^*−*^ cells and reduced activity of Ppt1 and CtsF. Intriguingly, α-mannosidase activity is also increased in *Mfsd8* ^*−/−*^ MEFs suggesting that the regulation of α-mannosidase may be an evolutionarily conserved function of MFSD8 (Danyukova et al., 2018). However, this effect may be cell type-dependent since Damme et al. (2014) reported unaltered activity of α-mannosidase in protein extracts generated from the cerebral cortex and liver of aged *Mfsd8*-depleted mice. In addition, a reduced amount of intracellular PPT1 was reported in human *MFSD8* knockout HAP1 cells (Danyukova et al., 2018). Finally, Danyokova et al. (2018) reported reduced amounts of intracellular CLN5 and CTSD, which is supported by our findings in *D. discoideum* of reduced intracellular levels of Cln5 and CtsD in *mfsd8* ^*−*^ cells due to increased secretion (Danyukova et al., 2018; Huber et al., 2020b). Combined, these findings support a role for MFSD8 in regulating lysosomal enzyme activity. However, at present, we are unable to determine if the altered activities of α-mannosidase, Ppt1, and CtsF in *mfsd8* ^*−*^ cells contributed to the delayed aggregation or if the delayed aggregation altered the activities of those enzymes.

Collectively, our data shows that Mfsd8 plays a pleiotropic role in regulating *D. discoideum* growth and the early stages of multicellular development. While the molecular function of Mfsd8 in *D. discoideum* is not known, several of the phenotypes we uncovered could be explained by Mfsd8 functioning as a chloride channel, as has been reported in mammalian models (Wang et al., 2021). Future research to resolve the molecular function of Mfsd8 in *D. discoideum* should provide clarity on how loss of *mfsd8* affects the diversity of processes we uncovered in this study. Intriguingly, aberrant phenotypes observed in cells lacking *mfsd8* are also seen in other *D. discoideum* NCL models, further suggesting that CLN proteins function in shared or convergent biological pathways (Journet et al., 1999; Huber et al., 2014; Huber, 2017; Huber et al., 2017; Huber and Mathavarajah, 2018; Mathavarajah et al., 2018; Smith et al., 2019; Huber, 2020; McLaren et al., 2021). Overall, the findings of this study have provided novel insight into the roles of Mfsd8 in *D. discoideum*, which could be used to inform research in mammalian models of CLN7 disease.

## Data Availability

The original contributions presented in the study are included in the article/Supplementary Material, further inquiries can be directed to the corresponding author.

## Abbreviations

AprA, autocrine proliferation repressor A; CadA, calcium-dependent cell adhesion protein A; cAMP, 3′,5′-cyclic adenosine monophosphate; CB, conditioned starvation buffer; CLN, ceroid lipofuscinosis neuronal; CM, conditioned growth media; CtnA, countin; CTSB, cathepsin B; CTSD, cathepsin D; CTSF, cathepsin F; DscA, discoidin A; FITC, fluoroscein isothiocyanate; HRP, horseradish peroxidase; MEF, mouse embryonic fibroblast; MES, 2-N-morpholinoethanesulfonic acid; MFSD8, major facilitator superfamily domain-containing 8; NAG, N-acetylglucosaminidase; NCL, neuronal ceroid lipofuscinosis; PPT1, palmitoyl-protein thioesterase 1; TPP1, tripeptidyl peptidase 1; WC, whole cell; WT, wild type.
