# Supplementary figures and images for: Mfsd8 Modulates Growth and the Early Stages of Multicellular Development in Dictyostelium discoideum
Source: Front Cell Dev Biol. 2022 Jun 9;10:930235. doi: 10.3389/fcell.2022.930235 (PMC9218796; doi:10.3389/fcell.2022.930235)

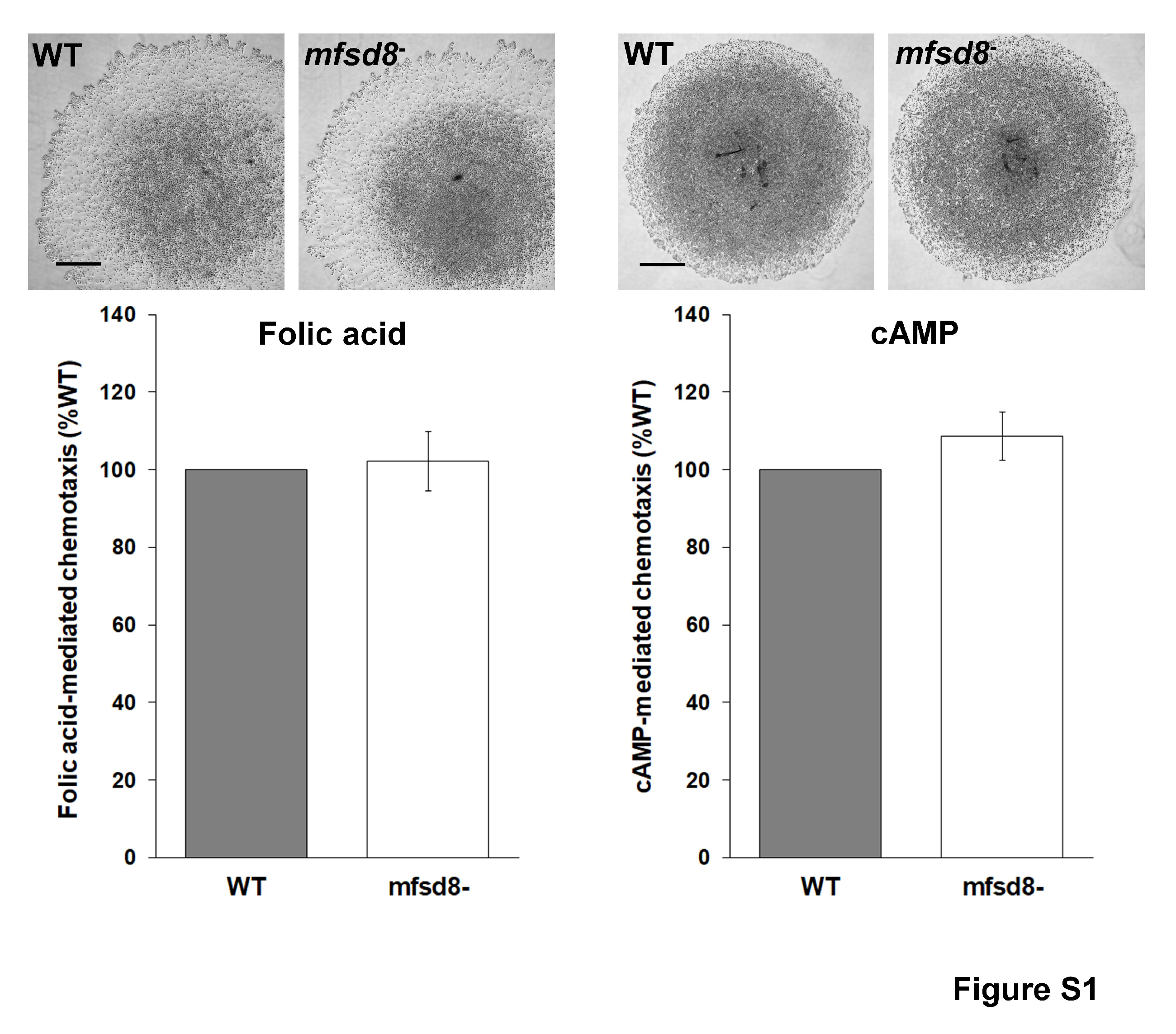

Supplement: Supplementary file 1 [file Image1.TIFF]
