# Supplementary material for: Mfsd8 Modulates Growth and the Early Stages of Multicellular Development in Dictyostelium discoideum
Source: Front Cell Dev Biol. 2022 Jun 9;10:930235. doi: 10.3389/fcell.2022.930235 (PMC9218796; doi:10.3389/fcell.2022.930235)
Supplement: Supplementary file 2 [file DataSheet1.docx]

**Supplemental methods**

**Radial bioassay of chemotaxis towards folic acid and cAMP**

Chemotaxis towards folic acid and cyclic adenosine monophosphate (cAMP) was assessed using a radial bioassay (O’Day, 1979; Huber and Mathavarajah, 2018b). Briefly, cells in the mid-log phase of growth were harvested from HL5, deposited into Petri dishes (5×10^6^ total cells), submerged in HL5, and incubated overnight at room temperature. The following day, confluent cells were harvested from the Petri dishes, washed two times with KK2 buffer, and plated (1×10^8^ cells/ml) in 0.6 μl volumes on 0.5% agar/KK2 ± folic acid (50 µM) or cAMP (10 μM). Cell spots were imaged at 0 and 5 hours using a Nikon Ts2R-FL inverted microscope equipped with a Nikon Digital Sight Qi2 monochrome camera (Nikon Canada Incorporated Instruments Division, Mississauga, Ontario, Canada). Images were viewed using NIS Elements Basic Research and analyzed using Fiji/ImageJ.

**Supplemental figure captions**

**Fig. S1. Effect of *mfsd8*-deficiency on folic acid-mediated and cAMP-mediated chemotaxis.** Cells in the mid-log phase of growth were deposited onto 0.5% agar/KK2 + folic acid (50 µM) or cAMP (10 μl). Images were taken once cells were deposited and after 5 hours. Scale bar = 500 μm. The amount of migration of each cell line was quantified using Fiji/ImageJ. Data presented as mean folic acid-mediated and cAMP-mediated chemotaxis ± SEM (n=8).

**References**

Huber, R. J., and Mathavarajah, S. (2018). Secretion and function of Cln5 during the early stages of *Dictyostelium* development. *BBA-Mol Cell Res.* 1865, 1437-1450.

O'Day, D.H. (1979). Aggregation during sexual development in *Dictyostelium discoideum*. *Can J Microbiol.* 25, 1416–1426.
